# Mr. Smith, on the Cutting Gorget

**Published:** 1809-09

**Authors:** Thomas Smith

**Affiliations:** Carlisle


### *To the Editors of the Medical and Physical Journal.*

Gentlemen,

PERMIT me to offer, through the medium of your very excellent Journal, a few remarks upon a paper by Mr. W. Simmons, of Manchester, published in the Edinburgh Medical Journal. This performance is entitled "*An Apology for the Cutting Gorget,*" and is *grafted,* as Mr. Simmons expresses it, upon "a *few* observations on Lithotomy, by Mr. Lawrence." At first sight of such a title, an advocate for the cutting gorget would naturally enough expect, that in the opinion of Mr. S. there were defects in that instrument to apologise for; such, for instance, as not effectually dividing the prostate; occasionally dipping into the rectum; or the like. This however is by no means the case. Mr. Simmons has no complaint whatever to prefer against the cutting gorget. He has found no defect in that instrument to apologize for; what is much more praise-worthy, in my opinion, the scope of Mr. Simmons's production, is to afford the whole professional body irrefragable proof, by twenty cases of lithotomy, performed during twenty years, that the gorget is a most perfect instrument; though not quite so easy to manage, by the bye, as Mr. Lawrence would have the world believe.

And here I may be allowed to digress, as a very material circumstance invites discussion, or at least, inquiry. This is, what description of proof Mr. Simmons's irrefragable proof is? If I rightly apprehend the question, it only amounts to this, that Mr. Simmons, "during twenty years of practice, has operated with the cutting gorget upon twenty patients labouring of stone, with so much success, as to render him satisfied with the instrument." Now it certainly is very comfortable for every operator to feel satisfied with his own performance, both in lithotomy and every other operation: but that any degree of satisfaction, or self complacency, can amount to irrefragable proof of any thing whatever, is somewhat questionable; and as Mr. Simmons has neither stated the proportion of successful cases, nor pointed out the obstacles he may have encountered, nor the accidents that may intervene, I am induced, for the sake of information, to make the inquiries, which will close my few observations. I do this with less hesitation, because I am confident, that the same frankness which has induced Mr. Simmons to come forward voluntarily tily to inform the professional world, will incline him to descend to such particulars as will supply all deficient information. Nor do I take an exception, that Mr. Simmons should choose for himself, success as a criterion of the fitness of that instrument. For although, in the same page, he attempts to detract from the merit of the celebrated Cheselden, by attributing the success of that great operator, not so much to the skilful operation he performed, as to his before unheard-of good fortune, "in meeting with so many patients labouring of stone, who were at the same time free from any disease of the kidneys, and whose habit did not predispose to inflammation or tetanic affection." Yet if Mr. Simmons with his favourite instrument, has equalled in success Mr. Cheselden with the knife, I shall consider such success as proof irrefragable in favour of the gorget, as far as twenty cases of lithotomy can make it so; all his patients having been free from disposition to tetanic affection, &c. &c. notwithstanding. But to proceed.

As it is my intention at some future period to avail myself of your useful Journal, in conveying to the public some remarks upon the defects, as well as excellencies of the cutting gorget, I shall excuse myself at present with this remark. Though I have used that instrument with as much success as my neighbours, and have felt confidence in it; yet when I reflect upon the great success of Cheselden's operation, and see, in the present day, anatomists of Mr. A. Cooper's eminence adopt the knife, after ample experience of the cutting gorget, I think it an example so well worthy of imitation, that I shall omit no opportunity of giving the knife a fair trial. Not however, in order that I may boast of my superior anatomical knowledge, or manual dexterity, but because I hope thereby to benefit my fellow creatures more effectually, and feel an ardent desire to imitate perfection whenever I discover it. For I am not, as Mr. Simmons expresses his feelings, "far from being inimical to improvement," but upon all occasions most anxious to adopt it. And I feel highly indebted to Mr. Lawrence for his candid and manly statement of a solitary case; concerning which, he has, in my opinion, left nothing to surmise; Mr. Simmons, therefore, has very prudently excused himself from hazarding his conjectures. In Mr. Lawrence's case, there is no concealment under an appearance of frankness. Mr. Lawrence gives a candid statement of the steps of an operation, whose success was complete, as far as respected the extraction of the stone; but its termination was unsuccessful, and he does not conceal it. In fact, Mr. Lawrence was not so fortunate in his subject for operation, as Mr. Cheselden is reported to have been; otherwise, it might have been suspected, that the integrity of the patient's constitution, not so much the skill of the operator, or superiority of the operation, was the cause of success.

I have already over-stepped the bounds which I had prescribed to my remarks, and shall therefore leave the remainder of Mr. Simmons's performance to the comments of Mr. Lawrence's abler pen; and hasten to make a few inquiries respecting the particular facts, the omission of which renders Mr. Simmons's proof incomplete. For this purpose, I would inquire, what proportion of unsuccessful cases have occurred in Mr. Simmons's practice? Has Mr. Simmons ever experienced difficulty in introducing the gorget, such as to oblige him to withdraw it, and make a second effort at introduction? Has Mr. Simmons ever experienced so much resistance to the extraction of a stone, as to require a considerable effort to overcome it? As Mr. Simmons reports, "that in twenty *different* instances, he has not had occasion to enlarge the wound in the bladder in *one single instance*," I beg leave to inquire the size of the largest stone Mr. Simmons has extracted? Under what circumstances would Mr. Simmons recommend the enlargement of the wound? and in what particular manner would he effect its enlargement? Has Mr. Simmons ever known on inspecting one of his patients after death, that the side of the bladder had been grazed to some depth by the cutting edge of the gorget? Upon what principle does Mr. Simmons prefer Hunter's gorget to the double cutting gorget of Mr. A. Cooper? If Mr. Simmons will oblige me by an answer to these inquiries, I shall feel great satisfaction; he will, I trust, cheerfully comply with my request; and when I shall have received this information, and have made a fair trial with the knife, I shall then think myself qualified to draw some valid comparison between the gorget and knife; and hope you will again permit me to avail myself of your excellent medium for publication.

I am, &c.

THOMAS SMITH.

*Carlisle, July* 10, 1809.

